# Persistent Corneal Epithelial Defects: A Review Article

**Published:** 2019

**Authors:** Uma Vaidyanathan, Grant C. Hopping, Harry Y. Liu, Anisha N. Somani, Yasmyne C. Ronquillo, Phillip C. Hoopes, Majid Moshirfar

**Affiliations:** 1 McGovern Medical School, Health Science Center, University of Texas, Houston, TX, USA; 2 Hoopes Durrie Rivera Research Center, Hoopes Vision, Draper, UT, USA; 3 John A. Moran Eye Center, Department of Ophthalmology and Visual Sciences, School of Medicine, University of Utah Salt Lake City, UT, USA; 4 Utah Lions Eye Bank, Murray, UT, USA

**Keywords:** Ophthalmic Solutions, Fibronectin, Thymosin Beta-4, Epidermal Growth Factor, Insulin-Like Growth Factor I, Growth Factor, Albumins

## Abstract

Persistent corneal epithelial defects (PEDs or PCEDs) result from the failure of rapid re-epithelialization and closure within 10-14 days after a corneal injury, even with standard supportive treatment. Disruptions in the protective epithelial and stromal layers of the cornea can render the eye susceptible to infection, stromal ulceration, perforation, scarring, and significant vision loss. Although several therapies exist and an increasing number of novel approaches are emerging, treatment of PEDs can still be quite challenging. It is important to treat the underlying causative condition, which may include an infection, limbal stem cell deficiency, or diabetes, in order to facilitate wound healing. Standard treatments, such as bandage contact lenses (BCLs) and artificial tears (ATs), aim to provide barrier protection to the epithelial layer. Recently-developed medical treatments can target the re-epithelialization process by facilitating access to growth factors and anti-inflammatory agents, and novel surgical techniques can provide re-innervation to the cornea. PEDs should be treated within 7-10 days to avoid secondary complications. These interventions, along with a step-wise approach to management, can be useful in patients with PEDs that are refractory to standard medical treatment. In this review, we discuss the epidemiology, etiology, diagnosis, current and novel management, and prognosis of persistent epithelial defects.

## INTRODUCTION

The cornea is the transparent, outermost layer of the eye that uniformly refracts the majority of light that enters the eye onto the lens and is essential for ideal vision. The multi-layered corneal epithelium acts as a protective barrier to infectious agents via tight junctions between neighboring cells, and it maintains its smooth optical surface by constantly regenerating cells in the basal cell layer [1, 2]. Disruptions in this protective layer can render the eye susceptible to infection, stromal ulceration, perforation, scarring, and decreased visual acuity [3-5]. The tear film, composed of lipid, aqueous, and mucin layers, also helps maintain corneal transparency and smoothness. For this reason, artificial tears are used as one of the initial therapies of ocular surface disorders to restore this film. Abrasions and corneal epithelial defects are some of the most common ocular pathologies that present to ophthalmologists. Persistent corneal epithelial defects (PEDs or PCEDs) result from the failure of rapid re-epithelialization and closure within 10-14 days after a corneal injury, even with standard supportive treatment [4, 5]. If left untreated, PEDs can result in significant complications, including infection and vision loss. This review of the current literature will encompass the epidemiology, etiology, diagnosis, current and novel management, and prognosis of persistent epithelial defects. 

**Epithelial Wound Healing**

The self-renewing corneal epithelium consists of stratified squamous cells and is separated into three layers: the outer two to three cell layers comprise the superficial layer, the middle two to three cell layers create the wing layer, and the innermost cell layer is the basal cell layer. The epithelial basement membrane resides between the basal epithelial cells and the stroma. The limbus, located at the periphery of the cornea, contains both epithelial stem cells, which proliferate into the basal epithelium, and basal cells. Epidermal growth factor (EGF) mediates the proliferation and migration process, as it promotes synthesis of nucleic acids in epithelial cells and stromal fibroblasts as well as stimulates the production of extracellular matrix protein, fibronectin, by epithelial cells. As basal cells migrate towards the central surface of the cornea, they gradually lose their proliferative properties and eventually undergo apoptosis and are desquamated into the tear film [4]. Several other growth factors, matrix and degradation proteins, and receptors are involved in the corneal healing process, making healing after an epithelial defect very complex. For example, metalloproteinases, cathepsins, and plasminogen are regulated by growth factors and play an important role in healing and tissue remodeling by modifying adhesion interactions and the extracellular matrix [6]. During corneal epithelial wound healing, inflammatory cytokines, such as tumor necrosis factor alpha (TNF-α) and interleukin-1 (IL-1), are released in response to damage to the epithelium. Keratocytes respond to IL-1 and produce growth factors, such as hepatocyte growth factor (HGF) and keratocytes growth factor (KGF), which influence the migration and proliferation of epithelial cells. Insulin-like growth factors (IGFs) and transforming growth factor beta (TGF-β) regulate differentiation and growth of stromal keratocytes and epithelial cells. Platelet-derived growth factors (PDGFs) regulate migration and proliferation of keratocytes, and thymosin-β4 promotes re-epithelialization and mediates epithelial migration during wound healing. Nerve growth factor (NGF) plays a vital role in trophic support, corneal sensation, and maintaining the tear film [4, 6]. Any alteration to this highly regulated process can result in abnormally slow healing. 

**Figure 1:**
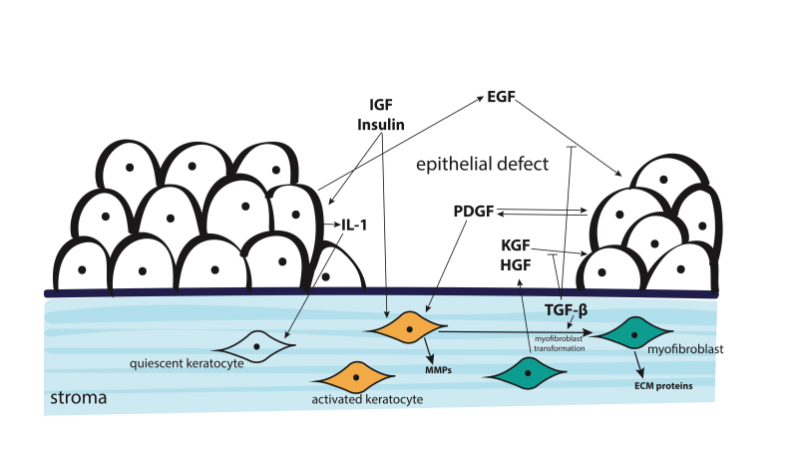
Growth Factors and Inflammatory Mediators Involved in the Epithelial Wound Healing Process. In the Case of Injury to the Cornea, Interleukin 1 (IL-1) is secreted by the Damaged Epithelial Cells, Causing some Keratocytes to Undergo Apoptosis and some to Proliferate into Activated Keratocytes. Epithelial Cells will also Secrete Transforming Growth Factor-beta (TGF-β) in Response to Destruction of the Basement Membrane and Results in Myofibroblast transformation. Growth Factors Insulin-like Growth Factor (IGF), Insulin, Epidermal Growth Factor (EGF), platelet-Derived Growth Factor (PDGF), Keratinocyte Growth Factor (KGF), and Hepatocyte Growth Factor (HGF) Play Important Roles in Corneal Wound Healing. EGF, IGF and Insulin Regulate Epithelial Growth and Stromal Keratocyte Activation. KGF and HGF are produced by Keratocytes to influence Migration and Proliferation of Epithelial Cells. PDGF Regulates Epithelial Proliferation and Keratocyte Function [2, 6].

**Figure 2:**
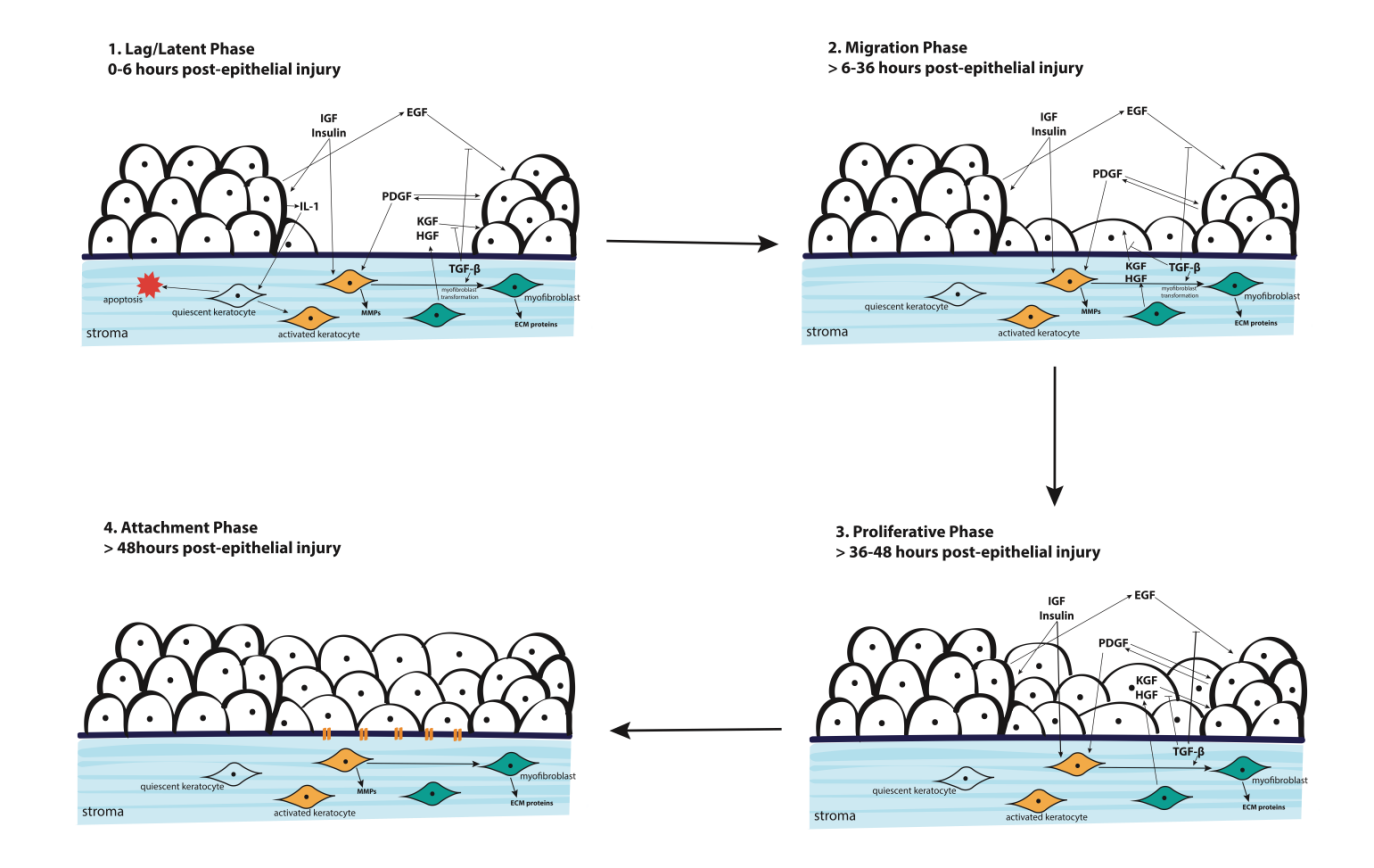
Normal Epithelial Wound Healing Process [11, 12].

**Figure 3:**
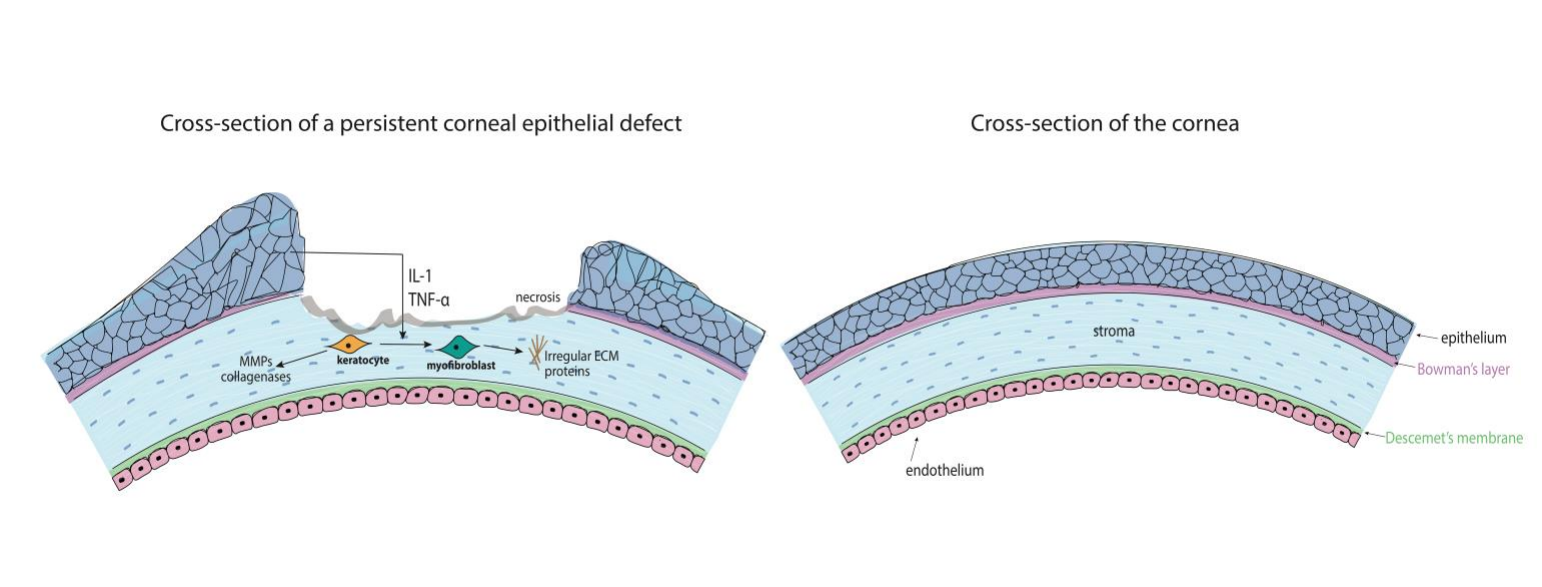
Depiction of a Persistent Epithelial Defect (PED). Note that in the Schematic of a PED, there is Loss of Part of the Anterior Stroma. Additionally, the Epithelial Cells are Unable to Migrate Centrally, Resulting in Epithelial Cell Growth on the Edges of the PED Lesion. The Basement Membrane has also been Eroded and Thinned. IL-1: Interleukin 1; TNF-α: Tumor Necrosis Factor; MMPs: Matrix Metallopeptidases; ECM: Extracellular Matrix

In normal conditions after an injury to the cornea, the epithelial layer undergoes an active repair process over 7-14 days, which involves a highly regulated cascade of growth factors, cellular signaling, proliferation, migration, extracellular matrix remodeling, and eventual quiescence or apoptosis [2, 4, 6]. Fig. 1 and 2 present the growth factors and inflammatory mediators involved in the normal corneal epithelial wound healing process. Only after re-epithelialization can the stroma adhere to the regenerated epithelial layer via hemidesmosomes anchoring to fibrils; therefore, if the epithelium cannot attach to the stroma, tissue defects can develop. If the underlying basement membrane remains intact, the epithelial healing process takes around 7 days to develop, regenerate, and adhere to the underlying basement membrane. However, if the underlying stromal layer is also affected in addition to the basement membrane, the epithelial layer will regenerate over the lesion and attach to the recovering stromal layer after around eight weeks. PEDs commonly extend into the stromal layer, causing stromal melting, secondary ulceration, and stromal scarring [4]. An insult to the cornea resulting in an *acute* epithelial defect is a lesion that usually heals over the 7-14-day time frame, whereas a *persistent* epithelial defect is unable to close within this normal interval. Fig. 3 models a persistent epithelial defect compared to the normal tissue layers of the cornea.

## METHODS

To find information on PED and current treatments, a literature search was performed using the following sources: PubMed, Google Scholar, Embase, and Scopus with the keywords “persistent epithelial defect”, “persistent corneal epithelial defect”, “PED”, “treatment of persistent epithelial defects”, “neurotrophic persistent corneal epithelial defect”, “non-healing corneal epithelial defect”, “corneal re-epithelialization”, “corneal wound healing”, and “epithelial abrasion”. Articles describing the epidemiology, etiology, diagnosis, current and novel management, and prognosis of PEDs were systematically reviewed. Reference lists detailed in each paper were examined to identify additional pertinent articles. There were no language restrictions. Publications were drawn between the dates of 1980-2019. 

**Epidemiology**

The incidence of PED is unknown. However, studies based on the etiology of the disease estimate that the annual incidence of PED is less than 200,000 cases in the U.S., thus classifying PED as a relatively rare disease [7]. For example, ocular herpes simplex can result in PED, epithelial and stromal keratitis, scarring, tissue destruction, neovascularization, and glaucoma. The incidence of ocular herpes simplex in the U.S. is an estimated 20.7 per 100,000 person-years. Additionally, in the U.S., the incidence of PED following a corneal transplant is around 7,558 cases per year, and the incidence of PED after a diabetic vitrectomy is around 2,480-5,257 cases per year. Diabetic keratopathy is estimated to occur in 47-64% of diabetic patients and increases the risk of acquiring epithelial defects [7]. 

A recent study on the epidemiology of epithelial defects after penetrating keratoplasty surgeries in patients with infectious keratitis reveals that factors that increase the risk for postoperative epithelial defects include male sex, > 60 years, graft diameter > 9 mm, bacterial or viral etiology, comorbid systemic diseases, such as rheumatic diseases and cancer therapy [8]. Additionally, another study showed that among a cohort of 53 children exposed to mechanical ventilation, 25% developed a corneal epithelial defect [9]. In addition, precautions and early management should be considered for patients who undergo pars plana vitrectomy due to the high incidence of postoperative PED [10].

**Etiology**

The normal corneal wound healing process can be disrupted from defective epithelial adhesion, limbal stem cell deficiency, surface trauma, medications, infections, and several other etiologies discussed below. The pathophysiology of PED involves proliferation of myofibroblasts and a resulting disordered extracellular matrix, producing an opacity in the cornea [5]. 

**Defective Epithelial Adhesion**

Basal epithelial cells produce and adhere to the basement membrane by forming hemidesmosomes and fibril connections. Defective epithelial adhesion or a deficient basement membrane can cause an increased risk for persistent corneal epithelial defects [4]. Recurrent corneal erosions can result in an overproduction of matrix metalloproteinases (MMPs) that disrupt the basement membrane and destroy fibril connections between the epithelium and basement membrane, leading to an increased risk for a PED. Epithelial basement membrane dystrophies (EBMD) can cause irregular extra basement membrane layers that are created and extend into the epithelium, resulting in a defective basement membrane and secondary adhesions. Toxic keratopathy can result in PEDs, as topical anesthetics can disrupt the migration of epithelial cells and interfere with hemidesmosome adhesion mechanisms between the epithelium and basement membrane. Degeneration of the cornea in Salzmann’s nodular degeneration, band keratopathy, bullous keratopathy, vitamin A deficiency, and scarring can result in PEDs due to abnormal formation of the basement membrane and defective adhesion mechanisms [4].

**Limbal Stem Cell Deficiency**

Epithelial cell migration and corneal epithelial stem cells in the limbus are crucial to regenerate the tissue after an insult to the corneal epithelium. Therefore, limbal stem cell deficiency (LSCD) often results in the inability of epithelial regeneration, stromal melting, scarring, persistent epithelial defects, corneal conjunctivalization, and neovascularization [13, 14]. One of the common causes of severe limbal stem cell deficiency is an alkali-induced chemical injury from lye, household cleaning solutions, and fertilizers, for example [15].

**Inflammation**

Cytokines, such as tumor necrosis factor alpha (TNF-α) and interleukin-1 (IL-1), mediate inflammation and induce production of growth factors by keratocytes that drive proliferation and migration of epithelial cells. These growth factors also promote the synthesis of proteases, such as MMPs and chemotactic factors that stimulate stromal remodeling and if in excess, stromal melting. Several inflammatory conditions can lead to PED due to disruption of corneal wound healing by over-activity of TNF-α, IL-1, and other inflammatory cytokines. These conditions include keratoconjunctivitis sicca, rosacea, infectious keratitis, autoimmune diseases, Sjögren’s syndrome, mucous membrane pemphigoid, Stevens-Johnson syndrome, graft versus host disease, peripheral ulcerative keratitis, Mooren’s ulcer, and rheumatoid arthritis [4].

**Neurotrophic**

The cornea is one of the most densely-innervated tissues in the human body and receives neural sensation and neurotrophic factors from the ophthalmic branch of the trigeminal nerve [16]. Neurotrophic persistent corneal epithelial defects are caused by local or systemic damage to the trigeminal nerve, resulting in decreased corneal sensation. The loss of corneal innervation causes degeneration of tissue, with the epithelium being most susceptible to damage, resulting in epithelial defects and poor corneal healing [16, 17]. Denervation causes decreased metabolism and proliferation of epithelial cells, resulting in subsequent epithelial layer dysfunction. Additionally, decreased blink rate, tear production, and Meibomian gland excretion can further exacerbate epithelial defects. Causes of damage to corneal nerves include diabetes mellitus, severe dry eye syndrome, current or past herpetic keratitis, anesthetic abuse, and traumatic or postoperative nerve damage [4, 16]. 

**Mechanical**

Recurrent abrasions and insults to the ocular surface can result in depletion of epithelial stem cells from a constant need to regenerate the epithelial layer. Thus, if the extent of loss of epithelial cells exceeds the healing properties of limbal stem cells, a PED can form. These mechanical injuries include exposure keratopathy from lagophthalmos, entropion or ectropion, trichiasis, blepharospasm, pseudomembranes or tarsal scars, and trachoma [4]. Severe dry eye disease can foster development of a PED, due to ocular surface mucin deficiency in the tear film from Stevens-Johnson syndrome or lacrimal gland damage in Sjögren’s syndrome, for example [7]. Additionally, a herpetic infection, dry eye disease, or inflammatory condition can cause mechanical irritation of the cornea from abnormal eyelids or even denervation of the cornea [4]. Corneal burns from chemical or thermal injuries can result in PED if the burn extends deep into the epithelium and stromal layer, resulting in an inability of re-epithelialization [18].

**Idiopathic and Hereditary Disorders**

Aniridia and corneal, stromal, and epithelial basement membrane dystrophies can cause PEDs, due to a deficiency in limbal stem cells and abnormal basement membrane adhesion mechanisms, respectively [4]. Table 1 highlights some of the common etiologies of PED.

**Table 1:** Etiologies of Defective Corneal Epithelial Wound Healing [4].

Underlying Etiology	Examples of causative diseases	Mechanism
Defective epithelial adhesion	Recurrent corneal erosions Epithelial basement membrane dystrophies (EBMD) Toxic keratopathy Salzmann’s nodular degeneration Band keratopathy Bullous keratopathy Vitamin A deficiency Scarring and trauma	Defective epithelial adhesion, deficient or abnormal basement membrane, overproduction of matrix metalloproteinases (MMPs), disruption of migration of epithelial cells
Limbal stem cell deficiency	Limbal stem cell deficiency (LSCD) Alkali-induced chemical injury Trauma	Deficiency of limbal stem cells
Inflammation	Keratoconjunctivitis sicca Rosacea Infectious keratitis Autoimmune diseases Sjögren’s syndrome Mucous membrane pemphigoid Stevens-Johnson syndrome Graft vs. host disease Peripheral ulcerative keratitis Mooren’s ulcer Rheumatoid arthritis	Over-activity of cytokines (TNF- α and IL-1), production of growth factors by keratocytes, proliferation and migration of epithelial cells, stromal remodeling
Neurotrophic	Diabetes mellitus Severe dry eye syndrome Current or past herpetic keratitis Anesthetic abuse Traumatic or postoperative nerve damage	Local or systemic damage to trigeminal nerve, loss of corneal innervation
Mechanical	Lagophthalmos Entropion or ectropion Trichiasis Blepharospasm Pseudomembranes or tarsal scars Trachoma Severe dry eye disease (mucin deficiency) Sjögren’s syndrome (lacrimal gland damage) Herpetic infection Chemical or thermal injuries resulting in corneal burns	Recurrent abrasions can result in depletion of epithelial stem cells, dry or inflammatory ocular surface, corneal erosions from eyelid abnormalities
Idiopathic and hereditary disorders	Aniridia Corneal, stromal and epithelial basement membrane dystrophies	Deficiency in limbal stem cells Abnormal basement membrane adhesion

TNF-α: Tumor Necrosis Factor Alpha; IL-1: Interleukin

**Diagnosis and Clinical Presentation**

Evaluating a PED involves fluorescein instillation to monitor the size, location, and depth of the defect [19]. In deeper PEDs, it takes a longer time for the fluorescein to absorb into the epithelium and stroma. A thorough physical exam should reveal findings such as inflammation in the anterior chamber, eyelid abnormalities, or decreased sensation of the cornea, such as in the case of a neurotrophic PED. 

Distinguishing an epithelial defect from a PED can be determined by the length of time the epithelial defect closes after injury to the cornea; an epithelial defect will recover after 7-10 days, whereas a PED will not heal even after 2 weeks and is usually refractory to standard therapies and supportive care [4]. A comprehensive patient history should be taken to highlight any possibilities of a previous herpetic infection, diabetes, immune system disorders, pain, or blurry vision. 

Although rare, LSCD commonly results in PED and may be clinically diagnosed by stippled late fluorescein staining and detection of corneal opacities, decreased vision, photophobia, unstable tear film, and superficial vascularization. PED along with LSCD may later present with scarring, ulceration, stromal neovascularization or perforation [20].

**Treatment of Persistent Epithelial Defects of the Cornea**

The consequences of an untreated PED include increased risk of infection, anterior stromal scarring, melting, neovascularization, ulceration, perforation, and significant vision loss [7]. Therefore, PEDs should be treated within 7-10 days to avoid secondary complications. Treatment should also increase in aggressiveness 10 days after diagnosis of a PED in hopes of preventing stromal fibrosis [5]. Clinicians should treat all cases of PEDs, including those with a partially healed epithelial layer. 

Management of PED is complex and involves several treatment options depending on its etiology. Healthy corneas may rapidly recover from an epithelial defect with supportive care; however, corneal healing may be hindered by disrupted cellular signaling from several etiologies that cause a PED, such as trauma, infection, severe dry eye disease, herpetic infections, diabetes mellitus, neurotrophic keratopathies, and complications after penetrating keratoplasties [3, 21]. PEDs occur if the cornea is unable to heal an epithelial defect within two weeks with standard supportive treatment; thus, the lesion exposes the underlying stromal layer to further damage, or infection [4]. Ocular epithelial diseases are particularly challenging to manage because treatment requires an understanding of the disease process and the mechanism of various medications and procedures discussed in this section. The goal of treatment is to provide protection and favorable conditions for the differentiated epithelial cells to migrate, proliferate, and regenerate the normal epithelial layer. Due to the complex nature of the disease, patients with PED must be thoroughly examined multiple times a week, or even daily, to ensure the effectiveness of the treatment course. Refer to Table 2 for a stepwise approach for the management of PED.

**Table 2:** Stepwise Approach for the Management of Persistent Corneal Epithelial Defects

Current Standard Management	Treatment of Refractory Cases	Novel treatments and therapies in development
**1. Treat underlying condition (i.e. diabetic keratopathies treated with optimal diabetes management; limbal stem cell deficiencies treated with limbal stem cell transplants; immunosuppression in Stevens-Johnson syndrome, graft vs. host disease, and Sjogren’s syndrome) ** **2. Consider iatrogenic causes (i.e. benzalkonium chloride, topical aminoglycosides, and vancomycin drops may disrupt epithelialization) ** **3. Aggressive lubrication with preservative-free artificial tears (ATs) and ocular ointments ** **4. Punctal plug ** **5. Bandage soft contact lenses (BCL) and/or pressure patching, although pressure patching may be less effective than BCLs** **6. Debridement, tarsorrhaphy, administration of botulinum toxin A, cyanoacrylates glue ** **7. Tetracyclines, prophylactic topical antibiotics and steroids (however, corticosteroids may cause stromal melting) ** **8. Refer to treatments of refractory cases**	**Medical**: Autologous serum eye drops Whole blood derived products (i.e. umbilical cord blood serum and platelet-rich fibrin tears) Scleral contact lenses, prosthetic replacement of the ocular surface ecosystem (PROSE) **Surgical**: Amniotic membrane grafting or transplant (AMT) with fibrin-glue or sutured underneath bandage contact lenses (BCL) Corneal epithelial stem cell transplantation Boston Keratoprosthesis (KPro) implantation Phototherapeutic keratectomy (PTK)	**Medical**: Topical fibronectin Topical thymosin beta 4 (Tβ4), topical fibronectin-derived peptide (PHSRN) Nexagon Topical Epidermal growth factor (EGF) Topical insulin-like growth factor-1 (IGF-1), topical insulin Human growth hormone (HGH) Albumin eye drops (AED) Matrix regenerating agent, ReGeneraTing Agent (RGTA) Amniotic membrane extract eye drops (AMEED) Oxervate (Cenergermin-bkbj), recombinant human nerve growth factor (rhNGF) **Surgical: ** Corneal neurotization

***Current Standard Management***

In approaching treatment of a PED, the first step is to consider the possible etiology of the disease along with its clinical presentation. For example, neurotrophic cornea can be treated with topical nerve growth factor; diabetic keratopathies with optimal diabetes management; herpetic keratitis with antiviral treatment and corticosteroids; and LSCD with limbal stem cell transplants [5].

The current standard management of PEDs includes a stepwise strategy, starting with conservative management and progressing to medical or surgical treatments if refractory. This includes lubrication, bandage soft contact lenses, punctal plugs, debridement, and tarsorrhaphy. Initially, aggressive lubrication every 1-2 hours with preservative-free artificial tears and ocular ointments are applied to the eye [3]. Iatrogenic causes of a PED must also be considered before proceeding to therapies after applying artificial tears and ointments. If the patient is concurrently using medications that may disrupt the corneal epithelium, such as benzalkonium chloride, topical aminoglycosides (i.e., gentamicin and tobramycin), and vancomycin drops, discontinuation of these medications may allow for re-epithelialization [3, 22].

The next step in treatment is the application of a punctal plug, which will increase retention of lubrication, which helps with normal corneal healing as tear film contains many growth factors including EGF, TGF, TGF-β, basic fibroblast growth factor (bFGF), HGF, calcitonin gene-related peptide, fibronectin, and vitamins A and C [23]. As discussed, it is important to recognize that any toxic medication that the patient is prescribed may be retained in the ocular surface with this procedure. Additionally, punctal occlusion may aggravate the corneal wound in patients with severe inflammatory disorders. Silicone or temporary collagen plugs are often used [3, 23].

Bandage soft contact lenses (BCL) along with preservative-free artificial tears and antibiotics are beneficial in protecting the damaged epithelium from mechanical erosion from eyelid blinking, thus aiding the re-epithelialization process. If stromal damage is involved, several weeks may be required for corneal healing. Lubrication and broad-spectrum topical antibiotics should be used concurrently to decrease the risk of infectious keratitis [24]. Patients with neurotrophic keratitis require consistent monitoring due to desensitization and increased risk of the progression of infection. Pressure patching is an alternative method to protect the cornea during re-epithelialization; however, pressure patching has been shown to impair wound healing and possibly be a source of infection, therefore requiring constant monitoring every 1-2 days [25]. Thus, pressure patching as an alternative to BCL may be less effective compared to the other therapies. 

Surgical interventions, such as debridement and tarsorrhaphy, are effective in most cases of PED refractory to medical management [3]. Debridement is the process of removing inert, healing epithelial tissue from the edge of the PED to allow for migration of new epithelial cells to restore the corneal tissue. The objective of a tarsorrhaphy procedure is to keep the palpebral fissure closed to decrease the area of exposed cornea. The drawstring temporary tarsorrhaphy is particularly useful in treating PEDs because it promotes easy access to the cornea for examination and medication delivery [26]. Temporary suture tarsorrhaphy is an option that may sustain the closed palpebral fissure for up to 6 weeks. Botulinum toxin A can be injected into the levator palpebrae superioris muscle to close the eyelids nonsurgically [3]. Temporary cyanoacrylate glue with patching therapy has been shown to help with the re-epithelialization process, prevent stromal melting, and has bacteriostatic activity [27].

Tetracyclines, prophylactic topical antibiotics, and steroids are also used as standard therapy for PED. Oral tetracyclines exhibit anticollagenolytic activity, inhibiting MMPs produced by inflammation mediators, and have been shown to be effective in healing PEDs within weeks [28]. Prophylactic topical antibiotics may be indicated when BCL or invasive procedures are used to prevent infection. Topical corticosteroids are controversial in treating PED because they may cause tissue destruction, stromal melting, and an increased risk of microbial keratitis [29]. However, corticosteroids can be very effective in treating persistent epithelial defects in herpetic keratitis, Stevens-Johnson syndrome, and atopic keratoconjunctivitis [5]. Immunosuppression is additionally effective in treating PED in Stevens-Johnson syndrome, graft vs. host disease, and Sjogren’s syndrome [5].

***Treatment of Refractory Cases***

Refractory cases of PED are those that do not respond to the standard therapies discussed above: lubrication, bandage soft contact lens, punctal plugs, debridement, and tarsorrhaphy. 

Amniotic membranes contain many of the growth factors (EGF, KGF, basic fibroblast growth factor [FGF2]), proteinase inhibitors and proteins that facilitate corneal wound healing in addition to providing a scaffold for re-epithelialization, decreasing vascularization, and having anti-inflammatory properties [3, 30, 31]. Therefore, amniotic membrane grafting or transplant (AMT) has proven to be extremely useful for reconstruction of surface ocular disorders, such as persistent epithelial defects and non-healing corneal ulcerations, to prevent corneal perforations [32-34]. Amniotic membranes are usually fibrin-glued or sutured underneath a bandage soft contact lens [3]. Amniotic membranes have also been indicated and used in the treatment of limbal stem cell deficiency to promote regeneration of the damaged epithelial layer [35, 36]. However, there are a few reported complications of AMT including postoperative infection, natural-occurring breakage of the sutures, and dissolution of the membrane, and hemorrhage beneath the amniotic membrane [7, 37]

Blood serum contains many growth factors, including vitamin A, vitamin E, EGF, TGF-β, PDGF, IGF, nerve growth factor, substance P, immunoglobulins, and fibronectin. Therefore, autologous serum eye drops are useful in re-epithelialization when a patient’s serum is diluted by 20% or 50% to create the eye drops and used every 3 hours daily [38-40] 50% autologous serum eye drops have been beneficial in treating severe ocular surface disorders, such as a PED [41, 42]. Both the combination of silicone-hydrogel soft contact lenses and autologous serum eye drops have been particularly successful in treating PEDs and preventing corneal stromal melting [38, 39, 43, 44]. However, there are a few limitations to using autologous serum, including the necessity of developing the tears from whole blood and associated regulatory restrictions [3].

Whole blood-derived products, such as umbilical cord blood serum and platelet-rich fibrin tears, can be used instead of autologous serum in patients with infection or systemic disease [3]. Patients treated with umbilical cord blood serum have been shown to have faster healing times for re-epithelialization than patients treated with platelet-rich fibrin tears [45]. Despite the benefit of whole blood-derived products in treating ocular surface diseases, these therapies are inconvenient due to the risks associated with the transport of whole blood-derived products, as well as the high supply costs [46].

Scleral contact lenses are effective in treating PED due to their high oxygen permeability, lubricating properties, and protective effects on the corneal epithelium [47]. Additionally, a prosthetic replacement of the ocular surface ecosystem (PROSE) can be used to promote healing overnight [48]. However, one complication of the therapies is microbial keratitis, warranting the need for prophylactic antibiotics [47, 48].

Transplantation of corneal epithelial stem cells can aid in re-epithelialization, especially in conditions that damage the limbal corneal stem cells, such as in Stevens-Johnson syndrome, chemical or thermal burns, or stem cell deficiencies [49, 50]. Severe cases of PED, such as patients with extensive alkali burns, can be treated with penetrating or lamellar keratoplasty if there is a high risk for perforation [51]. In patients with multiple failed corneal transplantations or grafts, the Boston Keratoprosthesis (KPro) implantation may help manage corneal LSCDs [52]. Phototherapeutic keratectomy (PTK) may be beneficial by applying a laser to the basement membrane and Bowman’s layer to facilitate stronger adhesion mechanisms [4, 53, 54]. PTK may be able to treat both refractive errors and epithelial defects [4].

***Novel Treatments***

The glycoprotein fibronectin is vital in cellular adhesion in the extracellular matrix. Thus, topical fibronectin may be helpful for re-epithelialization of a PED [55, 56]. Diabetic patients with PEDs experienced successful wound healing after topical therapy with fibronectin solution [57]. However, the benefit of topical fibronectin is controversial in treating persistent epithelial defects, as another study showed no significant improvement among patients with PEDs who received topical fibronectin [58].

Thymosin beta 4 (Tβ4) demonstrates efficacy in stimulating re-epithelialization processes and proliferation of epithelial cells while having anti-inflammatory effects [3]. The proposed mechanism of Tβ4 involves interfering with the synthesis of transcription factors mediating inflammation, such as nuclear factor kappa-light-chain-enhancer of activated B cells (NF-κB) and TNF-α [3, 59]. In addition, studies have shown the promising ability of Tβ4 as an anti-inflammatory agent to promote epithelial repair [59]. In a study with nine patients with chronic neurotropic PEDs, topical Tβ4 was demonstrated as a promising novel therapy to treat refractory neurotrophic corneal ulcers and epithelial defects [60]. A recently-developed topical fibronectin-derived peptide, PHSRN, was shown to facilitate wound healing of corneal epithelial defects in diabetic rat models [61]. 

Nexagon is an antisense oligodeoxynucleotide that downregulates connexin43 protein expression [62]. Nexagon shows therapeutic potential in treating severe ocular burns and epithelial defects with its ability to decrease edema and inflammation as well as promote cell proliferation [3, 63]. For example, in a prospective study of five eyes with PEDs, Nexagon was able to decrease inflammation and complete re-epithelialization in all five eyes [63].

***Therapies in Development***

Epidermal growth factor (EGF) stimulates proliferation of limbal and peripheral epithelial cells and stromal fibroblasts, promotes the synthesis of fibronectin, and serves as a chemotactic agent for both epithelial and stromal cells. Thus, EGF enhances the synthesis of extracellular matrix proteins to promote epithelial healing of corneal injuries [64]. Additionally, corneal wound healing is mediated by EGF-induced nuclear factor κB p50 and CTCF protein, making both mediators important in the growth factor-induced signaling pathway [65]. The EGF wound healing pathway also involves the activation of phospholipase D (PLD) and the downregulation of PAX6 expression [66, 67]. Topical EGF has been found to increase the rate of healing in corneal epithelial defects by almost twofold within one to two days [68, 69]. Another study on rabbit models demonstrated that the longer the epidermal growth factor is applied to corneal epithelial wounds, the faster the rate of epithelial wound healing [70]. In rabbit models, when EGF is applied in a controlled-release manner, corneal epithelial defects are found to heal significantly faster [71].

Insulin-like growth factor-1 (IGF-1) facilitates healing of PEDs by inducing migration and proliferation of corneal epithelial cells. Insulin, present in the tear film, also facilitates defect closure by promoting cell proliferation, as it is related to IGF-1 [72]. Additionally, IGF-1 stimulates expression of IGF receptors in limbal cells, promoting limbal stem cell differentiation [73]. Topical IGF-1, when applied with substance P, can improve corneal epithelial defects in patients with neurotrophic keratopathy by stimulating epithelial cell migration, limbal cell differentiation, and nerve regeneration in the cornea [7, 74]. Topical administration of substance-P-derived peptide with IGF-1 promoted resurfacing of PEDs within four weeks after initiating treatment in stem cell-positive patients with neurotrophic keratopathy [75]. Topical insulin is also involved in epithelial wound healing, as 0.5 units per drop four times per day is effective for healing postoperative corneal epithelial wounds in diabetic patients [76]. Using 1 unit of topical insulin four times a day postoperatively has also been shown to induce healing of corneal epithelial defects in diabetic patients after removal of epithelial tissue during vitreoretinal surgery [77]. In diabetic rats, insulin has helped promote re-epithelialization after corneal injury; thus, topical therapy utilizing insulin administration may be useful in treating diabetic keratopathies [78, 79].

Human growth hormone (HGH), normally produced by the anterior pituitary gland, potentially stimulates epithelial cells and fibroblast proliferation through an increase in phospho-STAT5 signaling. HGH may also stimulate corneal epithelial cell migration by an unknown mechanism [7]. However, in a recent study with mouse models, HGH has not shown a substantial effect on corneal wound healing [80].

Albumin eye drops (AED) may be effective in closing corneal epithelial defects. Albumin is the major protein component in autologous tears, and it, therefore, can be beneficial in treating ocular surface diseases such as corneal ulcers and epithelial defects [81, 82].

Matrix regenerating agent, also known as ReGeneraTing Agent (RGTA), includes polymers that are analogs to extracellular matrix proteins and act as a scaffold. RGTA also provides proteolytic defense for epithelial cells as they migrate and proliferate during corneal wound healing. Specifically, these analogs replace heparan sulfates and bind to collagen, elastin, and fibronectin to protect against proteolytic damage [83, 84]. In a recent study with twenty-one patients with PED, topical RGTA was shown to be effective in treating PEDs [85]. RGTA may be useful in managing neurotrophic keratopathy as well [84]. In a case study involving three patients with neurotrophic PEDs from distinct etiologies, RGTA eye drops administered with therapeutic contact lenses were beneficial in treatment after other conventional therapies were unsuccessful [83]. In another case study, three patients with PED were effectively treated with RGTA applied with BCL, resulting in re-epithelialization within 4-21 days [86]. 

Amniotic membrane extract eye drops (AMEED) can be utilized for tissue healing in severe cases of ocular damage. AMEED contains several diverse growth factors (EGF, HGF, bFGF, protease inhibitors, and the HC-HA/PTX3 complex), which supply AMEED with its growth-promoting and anti-inflammatory properties. Several studies have demonstrated the efficacy of AMEED therapy to improve ocular surface burns, ulcerations, and epithelial damage; therefore, AMEED appears to be a promising therapy in treating persistent epithelial defects [87]. Additionally, it has shown benefits in promoting proliferation of corneal epithelium *in vivo* and limbal stem cells *ex vivo* and *in vivo*; thus, AMEED may have potential use in treating LSCDs [88, 89]. 

Restoration of corneal sensation with corneal neurotization, a peripheral sensory nerve graft, is a recent and novel surgical technique to treat neurotrophic keratopathy. Corneal neurotization involves transferring the contralateral supraorbital and supratrochlear ophthalmic branches of the trigeminal nerve to the limbal cells of the cornea to re-innervate the corneal stroma and sub-basal layers [90, 91]. A recent study proposed, after *in vivo* confocal microscopic and histopathological analyses, that regeneration of corneal nerves works through a paracrine-mediated mechanism [92]. In a case study of six patients without corneal sensation, corneal neurotization was effective at restoring nerve function [93]. 

Oxervate medication, known as Cenegermin-bkbj or recombinant human nerve growth factor (rhNGF), has recently been FDA approved in the U.S. as a therapy for neurotrophic keratitis. Nerve growth factor restores sensation and sensitivity of the cornea, induces corneal healing, increases tear production, displays immunoregulatory effects, and plays a role in sensory and sympathetic nerve regeneration post-injury [94]. Phase I of the REPARO study determined that administering 10 or 20 ug/mL of rhNGF 6 drops per day for 8 weeks was well-tolerated and likely had little systemic absorption among patients with neurotrophic keratitis. Phase II of the study demonstrated in a multicenter, randomized, double-masked, and vehicle-controlled trial that treatment with topical rhNGF was not only well tolerated, but also effective in inducing corneal healing in patients with moderate-to-severe neurotrophic keratitis [95, 96]. 

## CONCLUSION

This review summarizes the epidemiology, etiology, diagnosis, clinical presentation, and management of persistent corneal epithelial defects. Clinicians should be aware that without treatment, a PED can cause stromal scarring, perforations, secondary infections, ulcerations, or even complete vision loss [3, 4]. Although several therapies exist, and an increasing number of novel approaches are emerging, treatment of PEDs can still be quite challenging. Standard treatments, such as BCLs and ATs, aim to provide barrier protection to the epithelial layer. More recently, medical treatments have been developed to target the re-epithelialization process by facilitating access to growth factors and anti-inflammatory agents, as well as new surgical techniques that can provide re-innervation to the cornea. Although PEDs refractory to treatment are common, newer interventions and a stepwise approach to management can aid in effectively promoting re-epithelialization in these patients.

## DISCLOSURE

Ethical issues have been completely observed by the authors. All named authors meet the International Committee of Medical Journal Editors (ICMJE) criteria for authorship of this manuscript, take responsibility for the integrity of the work as a whole, and have given final approval for the version to be published. No conflict of interest has been presented.

## Funding/Support:

Funding provided by Research to Prevent Blindness, NY, USA.
